# The antagonistic effect of FTO on METTL14 promotes AKT3 m^6^A demethylation and the progression of esophageal cancer

**DOI:** 10.1007/s00432-024-05660-2

**Published:** 2024-03-15

**Authors:** Ran Wei, Fangfang Zhao, Lingsuo Kong, Youguang Pu, Yuanhai Li, Chunbao Zang

**Affiliations:** 1 Department of Anesthesiology, The First Affiliated Hospital of Anhui Medical University, Hefei, 230001 Anhui People’s Republic of China; 2 Department of Anesthesiology, The First Affiliated Hospital of USTC, Division of Life Sciences and Medicine, University of Science and Technology of China, Anhui Provincial Cancer Hospital, Hefei, 230001 Anhui People’s Republic of China; 3 Department of Cancer Epigenetics Program, The First Affiliated Hospital of USTC, Division of Life Sciences and Medicine, University of Science and Technology of China, Anhui Provincial Cancer Hospital, Hefei, 230031 Anhui People’s Republic of China; 4 Department of Radiation Oncology, The First Affiliated Hospital of USTC, Division of Life Sciences and Medicine, University of Science and Technology of China, Anhui Provincial Cancer Hospital, Hefei, 230031 Anhui People’s Republic of China

**Keywords:** Esophageal cancer, *N*^6^-methyladenosine (m^6^a), FTO, METTL14, AKT3

## Abstract

**Background:**

As the most abundant modification in eukaryotic messenger RNAs (mRNAs), *N*^6^-methyladenosine (m^6^A) plays vital roles in many biological processes.

**Methods:**

Methylated RNA immunoprecipitation sequencing (MeRIP-seq) and transcriptomic RNA sequencing (RNA-seq) were used to screen for m^6^A targets in esophageal cancer cells and patients. The role of m^6^A RNA methylase in esophageal cancer was also analyzed using bioinformatics. In vitro and in vivo experiments were used to analyze gene expression and function. CCK-8, colony formation, cell apoptosis and immunofluorescence staining assays were performed to evaluate the proliferation, migration and invasion of esophageal cancer cells, respectively. Western blot analysis, RNA stability, RIP and luciferase reporter assays were performed to elucidate the underlying mechanism involved.

**Results:**

We found that the m^6^A demethylase FTO was significantly upregulated in esophageal cancer cell lines and patient tissues. In vivo and in vitro assays demonstrated that FTO was involved in the proliferation and apoptosis of esophageal cancer cells. Moreover, we found that the m^6^A methyltransferase METTL14 negatively regulates FTO function in esophageal cancer progression. FTO alone is not related to the prognosis of esophageal cancer, and its function is antagonized by METTL14. By using transcriptome-wide m^6^A-seq and RNA-seq assays, we revealed that AKT3 is a downstream target of FTO and acts in concert to regulate the tumorigenesis and metastasis of esophageal cancer. Taken together, these findings provide insight into m^6^A-mediated tumorigenesis in esophageal cancer and could lead to the design of new therapeutic strategies.

**Supplementary Information:**

The online version contains supplementary material available at 10.1007/s00432-024-05660-2.

## Background

The m^6^A modification of mRNAs was identified in the 1970s (Meyer and Jaffrey 2017; Balacco and Soller 2019), and m^6^A is the most abundant m^6^A modification in eukaryotic mRNAs and has unique distribution patterns (Holzer 1975). In mammalian cells, m^6^A modification is catalyzed by a methyltransferase complex (“writers”) composed of the proteins methyltransferase-like 3 (METTL3), METTL14, Wilms tumor 1-associated protein (WTAP), VIRMA (KIAA1429), and RBM15 (Sadecky et al. 1975; Poole-Wilson and Langer 1975). Notably, as the first identified RNA demethylase, fat mass- and obesity-associated protein (FTO) functions as an am^6^A “eraser” to remove m^6^A modifications from RNA, revealing that RNA modifications are reversible (Jia et al. 2011). Subsequently, the alkylation repair homolog protein 5 (ALKBH5) was proven to be another “eraser” of m^6^A modification (Bland et al. 1976), indicating the dynamic nature of m^6^A methylation. Since then, numerous studies have focused on the dynamics of m^6^A modification (Zhao et al. 2017a; Wang et al. 2014, 2015; Fu et al. 2015; Zhang et al. 2015). Moreover, m^6^A-binding proteins with a YTH domain, including the cytoplasmic proteins YTHDF1, YTHDF2, and YTHDF3 and the nuclear protein YTHDC1, have been identified as “readers” of m^6^A that modulate mRNA stability and translation (Zhou et al. 2015; Roundtree et al. 2017).

Recently, accumulated studies have focused on the biological functions of m^6^A modifications in mRNAs (Yue et al. 2015). It has been reported that m^6^A modification is involved in various biological processes, including the heat-shock response (Zhou et al. 2015), the DNA damage response (Xiang et al. 2017), mRNA clearance (Zhao et al. 2017a), neuronal functions (Lence et al. 2016), cortical neurogenesis (Yoon et al. 2017), progenitor cell specification (Zhang et al. 2017), and T-cell homeostasis (Li et al. 2017a). Moreover, m^6^A modification has been found to be associated with the tumorigenesis and progression of various cancers.

The m^6^A demethylase FTO was found to play critical roles in regulating fat mass, adipogenesis, and body weight (Fischer et al. 2009; Church et al. 2010; Merkestein et al. 2015). In addition, large-scale epidemiological studies have demonstrated the association of the FTO SNP risk genotype with the development of cancers such as breast, kidney, prostate, and pancreatic cancers, as well as leukemia, lymphoma and myeloma (Soderberg et al. 2009; Li et al. 2012; Hernandez-Caballero and Sierra-Ramirez 2015). Previous studies have shown that FTO plays a carcinogenic role in esophageal cancer and other solid tumors (Liu et al. 2020a; Niu et al. 2019; Guimaraes-Teixeira et al. 2021). A previous study showed that FTO plays an oncogenic role in cell transformation and leukemogenesis (Li et al. 2017b). However, the definitive role of FTO in cancer remains unclear.

In this study, we systematically investigated the role of m^6^A modification in the tumorigenesis of esophageal cancer, which is one of the most common forms of cancer worldwide (Kato and Nakajima 2013; Xu et al. 2020). We found a significantly upregulated level of FTO in esophageal cancer cells. Subsequent functional studies revealed an oncogenic role of FTO in esophageal cancer tumorigenesis. Moreover, we found that the m^6^A methyltransferase METTL14 is also involved in FTO-regulated m^6^A modification and acts in concert with FTO to regulate AKT3 methylation in the tumorigenesis and metastasis of esophageal cancer.

## Methods

### Samples, cell lines, and plasmids

Twenty-eight human esophageal cancer samples were obtained via abdominal surgery at the First Affiliated Hospital of University of Science and Technology of China, and the patients provided informed consent. The pathological condition was determined by an experienced surgical specialist. The esophageal cancer cell lines KYSE140 (BNCC351870), KYSE450 (BNCC339896), KYSE30 (BNCC339894) and KYSE150 (BNCC359343), and the human normal esophageal epithelial cell line HEEC (BRCC-003-0166) were obtained from the Chinese Academy of Cell Resource Center (Shanghai, China) (Zang et al. 2019).

Lentiviruses for overexpressing and silencing the expression of FTO, METTL14 and AKT3 were constructed by Hanbio Biotechnology Co., Ltd. Plasmids for the expression of Flag-tagged wild type (YTHDF1-WT, YTHDF2-WT, YTHDF3-WT, YTHDC1-WT, and YTHDC2-WT) were constructed with the p3xFLAG-*Myc*-CMV vector. For the shRNA plasmids used in lentivirus-mediated interference, complementary sense and antisense oligo nucleotides encoding shRNAs targeting YTHDF1 were synthesized, annealed and cloned and inserted into the pHBLV-U6-MCS-EF1-mcherry-T2A-PURO vector.

### Gene expression and survival analysis in esophageal cancer datasets

K‒M plotter (http://kmplot.com/analysis/) was used to assess the prognostic value of FTO and METTL14 expression in patients with esophageal cancer. The mRNA expression levels of FTO, YTHDC1 and YTHDF1 in cancer tissues and matched adjacent normal tissues of esophageal cancer patients were obtained from The Cancer Genome Atlas (TCGA) database. GEPIA2 (http://gepia.cancer-pku.cn/) was used to assess the correlation between FTO and AKT3.

### m^6^A content analysis

The EpiQuikTM m^6^A RNA Methylation Quantification Kit (Epigentek) was used to analyze the content of m^6^A in total RNA (Cheng et al. 2019).

### m^6^A RT–PCR

According to a previously described protocol, m^6^A-RT–PCR was conducted (Cheng et al. 2019). To obtain the m^6^A pull-down region, 2 μg of RNA was immunoprecipitated with the m^6^A antibody in 500 μl of IP buffer. m^6^A RNA was immunoprecipitated with Dynabeads^®^ Protein A and then eluted twice with elution buffer. m^6^A IP RNA was recovered by ethanol precipitation. Then, 2 ng of total RNA and m^6^A IP RNA were used as templates for qRT–PCR.

### RNA pull-down assays

RNA was extracted from the Megascript^®^ T7 Transcription Kit (Ambion) through the In Vitro Transcription Kit, and the Pierce Magnetic RNA‒Protein Pull-Down Kit (Thermo Scientific) was subsequently used to conduct the RNA pull-down experiment. In brief, a Pierce RNA 3′ End Desthiobiotinylation Kit (Thermo Scientific) was used to biotinize the RNA. Then, 50 pmol of biotinylated RNA, 50 μl of streptavidin magnetic beads and 200 μg of cell lysates were incubated at a suitable temperature for a certain period of time, and the supernatant was collected after repeated washing for real-time PCR and Western blotting analysis.

### RIP assays

A Magna RIP RNA-Binding Protein Immunoprecipitation Kit (Millipore) was used for RIP according to the manufacturer’s instructions. Briefly, KYSE150 cells were lysed before centrifugation, incubated with magnetic beads and coated with antibodies for 4 h or overnight at 4 °C. Then, the complexes were washed and incubated with proteinase K. The samples were centrifuged and placed on a magnetic separator, and the supernatants were used to extract RNA with an RNA extraction kit (Bioline) (Sang et al. 2018).

### m^6^A-seq and data analysis

Total RNA was isolated. RNA fragmentation, m^6^A-seq and library preparation were performed according to the manufacturer’s instructions (Ping et al. 2014). An RNA Library Prep Kit (NEB, USA) was used for library preparation. The m^6^A-seq data were analyzed according to the manufacturer’s protocols.

### Vector and m^6^A mutation assays

The potential m^6^A sites in the full-length AKT3 transcripts were predicted using the online tool SRAMP (http://www.cuilab.cn/sramp/). The m^6^A motif-depleted 3′UTR regions were cloned and inserted into pGL3 for luciferase reporter gene analysis.

### RNA stability

Actinomycete D (6 μg/ml)-treated downregulated FTO cells and control cells were used to block RNA transcription at 0, 2, 4, 6, and 8 h. The AKT3mRNA residue was detected by qPCR, after which the stability of the mRNA was calculated.

### Luciferase reporter assays

Cells were transfected with pGL3, pGL3-WT-3′UTR, pGL3-Mut1-3′UTR or pGL3-Mut2-3′UTR in a six-well plate. After transfection for 8 h, each cell line was reseeded into a 96-well plate. After 24 h of incubation, both firefly and Renilla luciferase activities were measured 24 h after transfection using the Dual-Luciferase Reporter Assay System (Promega) (Pu et al. 2017).

### In vivo xenograft model

For the subcutaneous transplantation model, cells were diluted in 100 μl of PBS + 100 μl of Matrigel (BD) and subcutaneously injected into immunodeficient male mice to investigate tumor growth. When the tumor volume in each group reached ~100 mm^3^, the mice were anesthetized with a small flow of carbon dioxide until they were unconscious. Then, the mice were killed completely. Then, the tumors were removed and weighed for use in immunohistochemistry assays and further studies.

For the in vivo lung metastasis model, mice were injected with WT (wild type), sh-FTO, AKT3-OE or sh-FTO + AKT3-OE KYSE150 cells (1 × 10^6^ per mouse, *n* = 3 for each group). Six weeks after injection, the mice were killed, and metastatic lung tumors were analyzed.

### Immunohistochemistry assays

Tissue arrays were constructed using 28 pairs of esophageal cancer and paracancerous tissues as well as animal experimental specimens. Paraffin embedding, sectioning and immunohistochemistry were used to detect FTO expression in esophageal cancer and paracancerous tissues and vimentin, E-cadherin and MMP2 expression in animal specimens.

### Statistical analysis

Microsoft Excel software and GraphPad Prism were used to assess the differences between the experimental groups. Statistical significance was analyzed by a two-tailed Student’s *t* test and one-way ANOVA. *p* values less than 0.05 were considered to indicate statistical significance: *, *p* value <0.05; **, *p* value <0.01; and ***, *p* value <0.001.

## Results

### FTO negatively correlates with METTL14 in esophageal cancer cells

**Fig. 1:**
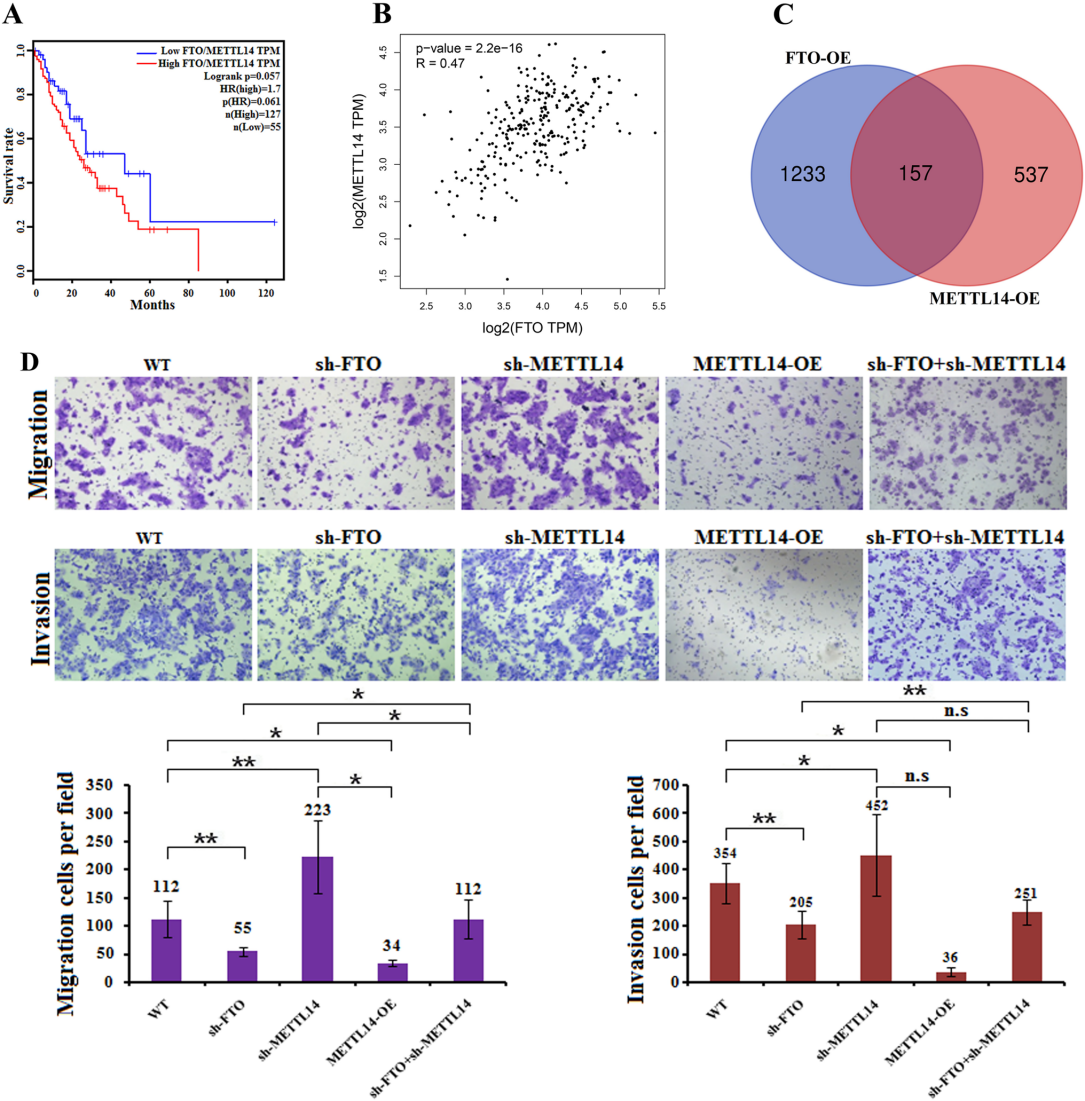
Correlation analysis of FTO and METTL14. **A** K‒M survival analysis of patient OS according to the ratio of FTO mRNA expression to METTL14 expression in esophageal cancer tissues; the greater the ratio was, the worse was the prognosis. **B** The abundance of m^6^A on AKT3 mRNA transcripts in KYSE30 and KYSE150 cells, as detected by m^6^A-seq, was plotted using an Integrative Genomics Viewer (IGV). The *y*-axis shows the sequence read number, the blue boxes represent exons and the blue lines represent introns. Reduction in m.^6^A modifications in specific regions of AKT3. **C** Venn diagram analysis showing that the intersection genes overexpressed FTO (FTO-OE) in KYSE150 cells and overexpressed METTL14 (METTL14-OE) in KYSE150 cells. **D** FTO knockdown in KYSE150 cells decreased migration and invasion, while METTL14 knockdown (sh-METTL14) or overexpression (METTL14-OE) inhibited or restored FTO function, respectively. The ability of the cells was assessed by Image-Pro Plus 6.0 software. n.s., not statistically significant; *, *p* value < 0.05; **, *p* value < 0.01

Our research showed that the independent expression of FTO was not significantly correlated with the prognosis of esophageal cancer patients, which is different from the mechanism of ALKBH5 (Fig. S1A, B) (Kong et al. 2023). Analysis of esophageal cancer patients with higher FTO/METTL14 ratios revealed poorer prognoses than patients with lower FTO/METTL14 ratios (Fig. 1A, B). Further analysis of the differentially expressed genes by RNA-seq revealed that FTO overexpression and METTL14 overexpression resulted in a total of 157 shared differentially expressed genes (Fig. 1C). These results indicated that FTO might be functionally associated with METTL14. To further examine the correlation between FTO and METTL14, we evaluated the ability of KYSE150 cells to proliferate and reverse changes in FTO or METTL14 expression. As expected, downregulation of FTO or upregulation of METTL14 reduced proliferation, which could be restored via downregulation of METTL14. Moreover, the decreased migration or invasion of KYSE150 cells induced by FTO knockdown could also be restored by METTL14 overexpression. In contrast, METTL14 knockdown had the opposite effect: METTL14 knockdown increased the migration or invasion of KYSE150 cells, but the effects of METTL14 on esophageal cancer cell phenotype induced by FTO between the two (Fig. 1D). These results suggest that FTO and METTL14 negatively correlate with each other and function as biomarkers in esophageal cancer patients. Moreover, the above results indicate that FTO promotes invasion, metastasis, and proliferation in esophageal cancer. However, during the development of esophageal cancer, FTO has an antagonistic effect on the expression of METTL14, and this effect is related to the ratio of FTO to ALKBH5.

### FTO is involved in the proliferation, migration, invasion, and apoptosis of esophageal cancer cells

**Fig. 2:**
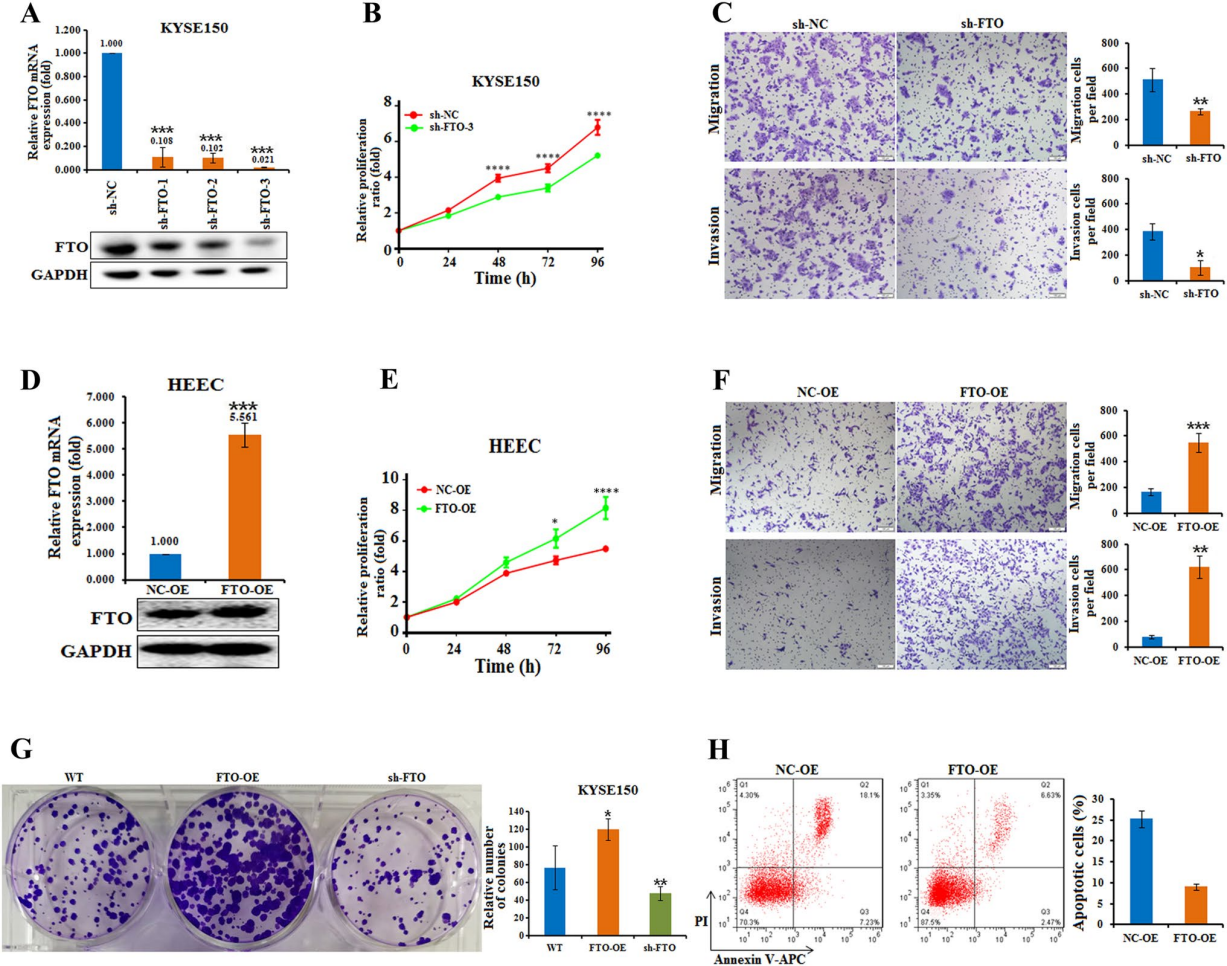
Effect of FTO on esophageal cancer cell proliferation, migration, invasion, apoptosis and colony formation ability. **A** The effect of specific shRNAs (sh-FTO-1, -2 and -3) on KYSE150 cells was verified at both the mRNA (by qRT‒PCR) and protein (by western blot) levels. ***, *p* value < 0.001. **B** CCK-8 assays every 24 h showed that FTO knockdown inhibited the proliferation of KYSE150 cells compared with that of the negative control (sh-NC). ***, *p* value < 0.001. **C** FTO knockdown in KYSE150 cells decreased migration and invasion compared to those of the negative control (sh-NC). The invasive ability of the cells was assessed by Image-Pro Plus 6.0 software. *, *p* value < 0.05; **, *p* value < 0.01. **D** The levels of FTO in HEEC cells transfected with FTO-OE or the negative control (NC-OE) were measured by real-time PCR and western blot analyses and are shown. n.s., not statistically significant; *, *p* value < 0.05; **, *p* value < 0.01. **E** CCK-8 assays every 24 h showed that FTO overexpression promoted the proliferation of HEECs compared with that of the negative control (NC-OE). n.s., not statistically significant; *, *p* value < 0.05; ***, *p* value < 0.001. **F** Compared with the negative control (NC-OE), FTO-overexpressing HEECs increased migration and invasion. The invasive ability of the cells was assessed by Image-Pro Plus 6.0 software. **, *p* value < 0.01; ***, *p* value < 0.001. **G** Colony formation assays showed that FTO promoted cell proliferation in KYSE150 cells treated with FTO-expressing lentivirus (FTO-OE) or with an FTO knockdown vector (sh-FTO) compared with wild-type (WT) cells. **H** FTO overexpression in HEECs decreased apoptosis, as determined by Annexin V/PI staining and FACS. The quantification of apoptotic cells was performed, and the numbers represent the sum of early and late apoptotic cells

Experiments have shown that FTO is significantly upregulated in esophageal cancer cell lines and patient tissues (Fig. S1C–F). To investigate the potential roles of FTO in esophageal cancer cells, we performed a series of functional assays to characterize the effect of FTO. We first downregulated the expression of FTO by transfecting KYSE150 cells with FTO shRNAs (Fig. 2A). Subsequent CCK-8 assays showed that downregulation of FTO expression significantly inhibited the proliferation of KYSE150 cells (Fig. 2B). Moreover, FTO knockdown in KYSE150 cells decreased cell migration and invasion compared to those of the control cells (Fig. 2C). Previous studies have shown that E-cadherin is a biomarker for cell migration (Lin et al. 2019; Petrova et al. 2016); thus, we detected the expression of E-cadherin in FTO-knockdown cells (Fig. S2A). The results showed that downregulation of FTO was correlated with increased expression of E-cadherin (E-cad) in KYSE150 cells, indicating that FTO might be associated with E-cadherin-regulated cell migration in esophageal cancer cells.

Afterwards, we overexpressed FTO in HEECs by transfection of FTO-overexpressing lentivirus (Fig. 2D). The cell proliferation rate was significantly increased upon overexpression of FTO in HEECs (Fig. 2E). Moreover, cell migration and invasion were also increased in FTO-overexpressing HEEC cells (Fig. 2F). In addition, the colony formation assays showed that, compared to that in the control cells, FTO overexpression significantly promoted cell proliferation in KYSE150 cells, whereas FTO knockdown largely impeded cell proliferation (Fig. 2G). Moreover, compared to that in the control cells, the percentage of apoptotic HEECs strongly decreased from 25 to 10%, as determined by flow cytometry, indicating that the percentage of apoptotic cells decreased with increasing FTO overexpression (Fig. 2H). All these results indicated that the oncogenic role of FTO is involved in cell proliferation, migration, invasion, and cell apoptosis in esophageal cancer cells.

### AKT3 is regulated by FTO-mediated m^6^A modification in esophageal cancer cells

**Fig. 3:**
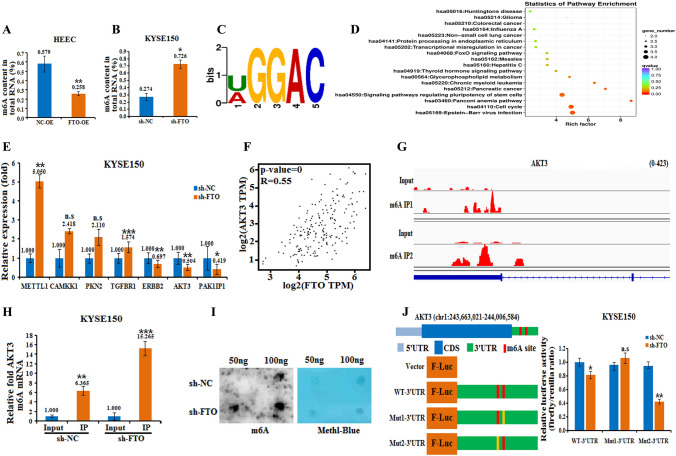
Identification of potential targets of FTO in esophageal cancer cells via transcriptome-wide m^6^A-seq and RNA-Seq assays. HEEC and KYSE150 cells were subjected to FTO overexpression (FTO-OE) (**A**) or knockdown (sh-FTO) (**B**) treatment, and the m^6^A content of the total mRNA was determined with an m^6^A RNA Methylation Quantification Kit. **C** The motif of FTO was analyzed with the RMBase V2.0 database (http://rna.sysu.edu.cn/rmbase/). **D** A clusterProfiler was used to identify the enriched KEGG pathways associated with 128 genes, which showed 1.5-fold greater m^6^A expression upregulation in esophageal cancer cells than in control cells. **E** The mRNA levels of the six differentially expressed genes METTL1, CAMKK1, PKN2, TGFBR1, ERBB2,AKT3 and PAK1P1 in KYSE150 cells with FTO knockdown versus the negative control (sh-NC) were measured by real-time PCR. n.s., not statistically significant; *, *p* value < 0.05; **, *p* value < 0.01; ***, *p* value < 0.001. **F** The interaction between FTO and the AKT3 gene was analyzed using the GEPIA database. **G** The abundance of m^6^A on AKT3 mRNA transcripts in KYSE30 and KYSE150 cells, as detected by m^6^A-seq, was plotted using an Integrative Genomics Viewer (IGV). The *y*-axis shows the sequence read number, the blue boxes represent exons, and the blue lines represent introns. Reduction in m^6^A modifications in specific regions of AKT3. **H** Detection of m^6^A methylation levels in AKT3 *cells* by MeRIP-PCR with an m^6^A RNA Methylation Quantification ELISA Kit. **I** Dot plot analysis of the overall level of m^6^A modification in esophageal cancer cells before and after knockdown of the FTO m^6^A modification site. **J** Schematic representation of the positions of m^6^A motifs within the AKT3 mRNA transcript and the 3′UTR mutation (GGAC to GGCC) in the pmirGLO vector used to investigate the role of m^6^A in regulating AKT3 expression. pmirGLO-WT-3′UTR or pmirGLO-Mut1/2–3′UTR reporters were transfected into KYSE150 cells with FTO knockdown versus the negative control (sh-NC), after which the relative luciferase activity was measured

To investigate the potential role of FTO in tumor progression, we detected the m^6^A content in total mRNA with anEpiQuik™ m^6^A RNA Methylation Quantification Kit (colorimetric) in FTO-overexpressing HEEC and FTO-silenced KYSE150 cells. As expected, FTO overexpression significantly decreased the m^6^A content in HEECs (Fig. 3A), whereas FTO silencing dramatically increased the m^6^A content in KYSE150 cells (Fig. 3B). As analyzed by the RMBase database (https://rna.sysu.edu.cn/rmbase/), the genes with m^6^A modification had a consensus motif of U/AGGAC, which is a common feature among the genes with m^6^A methylation (Fig. 3C).

We took the intersection of the downregulated peak after overexpression of FTO and the upregulated peak after interference with FTO and then enriched the KEGG function of the intersected genes. The results showed that the cell cycle and other pathways were significantly enriched for the genes with different peaks. Compared to those in control cells, 128 genes exhibiting a 1.5-fold change in m^6^A expression were identified in esophageal cancer cells. Kyoto Encyclopedia of Genes and Genomes (KEGG) enrichment analysis indicated that a handful of genes were associated with the different metabolic pathways in various cancer cells (Fig. 3D). Overall, the analysis of m^6^A modifications after FTO knockout indicated that FTO methylation was related to m^6^A methylation. Among these genes, we selected the top seven most differentially expressed genes and detected their expression in KYSE150 cells by real-time PCR. The results showed that METTL1, CAMKK1, PKN2, and TGFBR1 were upregulated in FTO-silenced KYSE150 cells, whereas ERBB2, AKT3 and PAK1P1 were downregulated (Fig. 3E). Among the three downregulated genes, AKT3, which is a serine/throne kinase from the AKT family, is involved in the biogenesis of many different types of cancers (Liu et al. 2018).

The correlation prediction of FTO and AKT3 by the GEPIA database yielded an Rvalue of 0.55, which strongly indicated the physical interaction between FTO and the AKT3 gene (Fig. 3F). We thus detected the abundance of m^6^A on AKT3 mRNA transcripts in KYSE30 and KYSE150 cells by m^6^A-seq, and the results showed that m^6^A methylation was enriched in the exons and 3′UTRs of AKT3, with a clustered distribution (Fig. 3G).

To further investigate the role of AKT3 in FTO-regulated m^6^A methylation, we detected the m^6^A content in KYSE150 cells by MeRIP-PCR. The results showed that FTO knockdown retained m^6^A methylation in AKT3 cells, as shown by the elevated m^6^A content in KYSE150 cells (Fig. 3, H, I). To examine the role of m^6^A methylation on the AKT3 3′UTR, firefly luciferase reporters were generated, followed by the wild-type AKT3 3′UTR, mutant1 or mutant2 3′UTR. 3′UTR reporter luciferase assays showed that, compared to those in control cells, the 3′UTR of the mutant1 gene slightly but not significantly reduced AKT3 expression, whereas the 3′UTR of the mutant2 gene significantly suppressed AKT3 expression (Fig. 3J). The results indicated that m^6^A methylation of the 3′UTR might be involved in m^6^A modification-regulated AKT3 expression.

### AKT3 is involved in m^6^A-regulated esophageal cancer tumorigenesis and metastasis

**Fig. 4:**
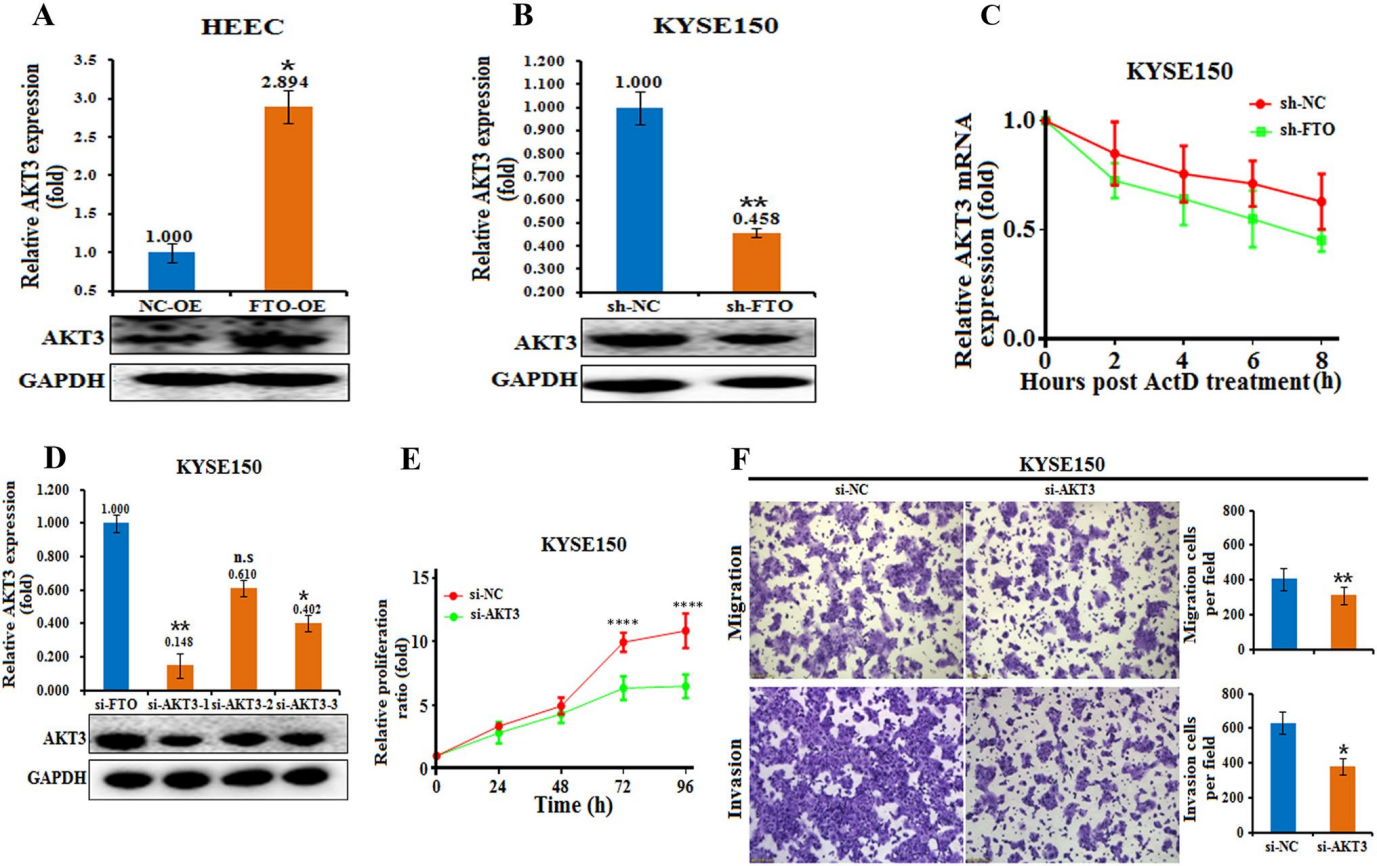
AKT3 is a critical target of FTO that mediates esophageal cancer cell growth, survival and invasion. **A** The levels of AKT3 in HEECs transfected with FTO-OE or the negative control (NC-OE) were measured by real-time PCR and western blot analyses and are shown. *, *p* value < 0.05. **B** The levels of AKT3 in KYSE150 cells with FTO knockdown versus the negative control (sh-NC) cells measured by real-time PCR and western blot analyses are shown. **, *p* value < 0.01. **C** The mRNA level of AKT3in KYSE150 cells with FTO knockdown and then treated with Actinomycete D (6 μg/ml) for 0, 2, 4, 6, or 8 h was measured by real-time PCR. **D** The effect of specific siRNAs (si-AKT3-1, -2 and -3) on KYSE150 cells was verified at both the mRNA (by qRT‒PCR) and protein (by western blot) levels. n.s., not statistically significant; *, *p* value < 0.05; **, *p* value < 0.01. **E** CCK-8 assays every 24 h showed that AKT3 knockdown inhibited the proliferation of KYSE150 cells compared with that of the negative control (sh-NC). ***, *p* value < 0.001. **F** Compared with the negative control (si-NC), AKT3 knockdown in KYSE150 cells decreased migration and invasion. The invasive ability of the cells was assessed by Image-Pro Plus 6.0 software. *, *p* value < 0.05; **, *p* value < 0.01

We then characterized the roles of AKT3 in esophageal cancer cell functions by several in vitro experiments. Notably, overexpression of FTO in HEEC cells largely increased the expression of both AKT3 mRNA and protein, whereas FTO knockdown in KYSE150 cells decreased the expression of AKT3 mRNA and protein (Fig. 4A, B). To further investigate whether FTO affects the stability of AKT3 mRNA, we tested AKT3 mRNA levels in KYSE150 cells with FTO knockdown after treatment with actinomycete D, which is a metabolic inhibitor (Fig. 4C). The results showed that the mRNA level and stability of AKT3 dramatically decreased over time with FTO knockdown, indicating that FTO might increase the stability of AKT3 mRNA, which results in a greater level of AKT3 protein expression.

Next, we downregulated AKT3 expression in KYSE150 cells and tested its effect on KYSE150 cell functions. Transfection of KYSE150 cells with one of the three AKT3 siRNAs significantly decreased the expression of AKT3 at both the mRNA and protein levels (Fig. 4D). In addition, the overexpression of AKT3 also increased the expression of vimentin, indicating that AKT3 promotes tumor cell migration (Fig. S2B). Along with the decrease in AKT3 expression in KYSE150 cells after si-AKT3 transfection, the cell proliferation ratio also decreased over time (Fig. 4E). Moreover, cell invasion and migration were also decreased with AKT3 knockdown in KYSE150 cells (Fig. 4F). These results indicated that AKT3 is involved in the tumorigenesis of esophageal cancer progression.

### FTO and AKT3 act in concert to regulate esophageal cancer cell tumorigenesis and metastasis

**Fig. 5:**
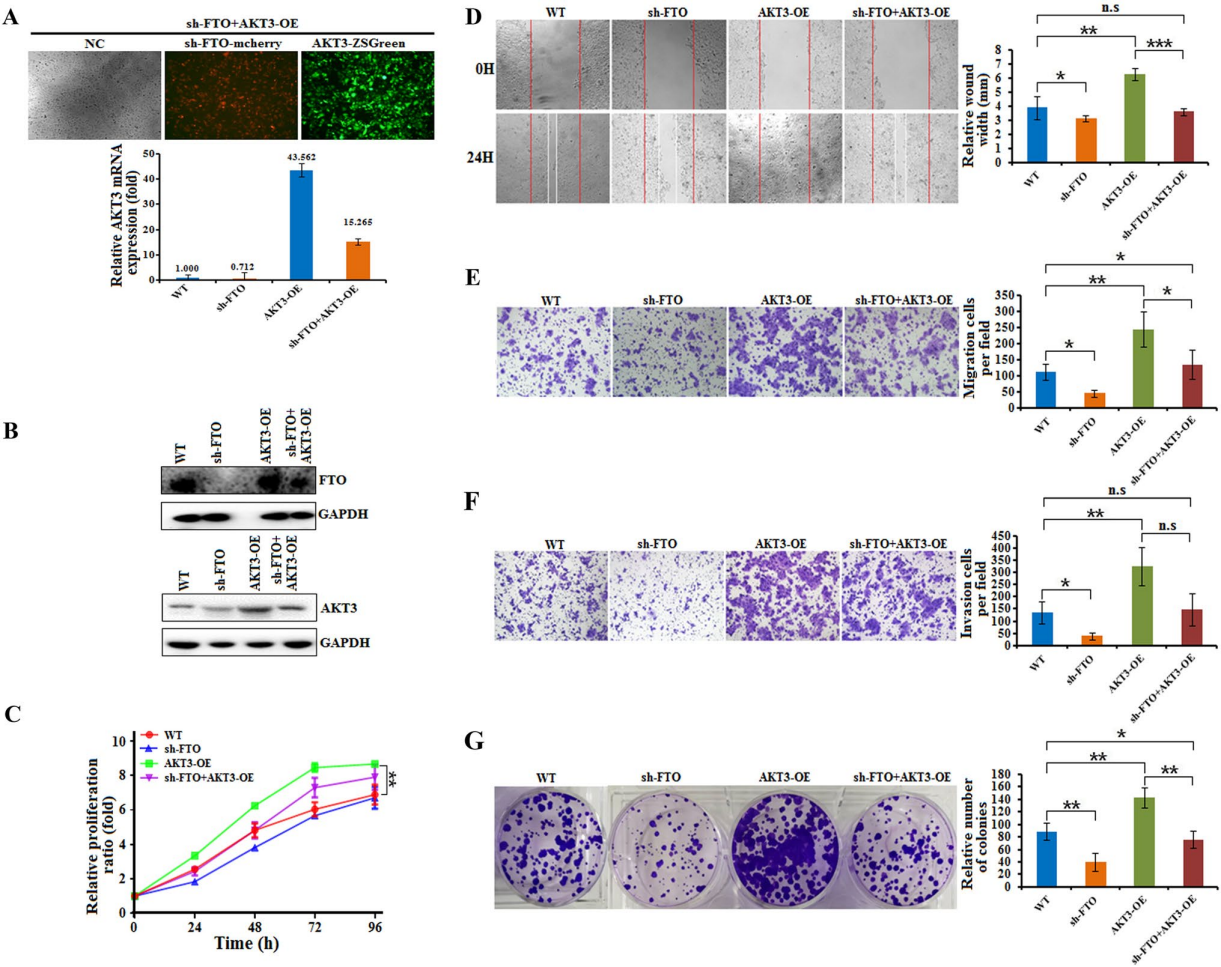
The functions of FTO and AKT3 in esophageal cancer cells are mutually restricted. **A** The relative AKT3 mRNA expression levels in KYSE150 cells infected with FTO knockdown lentivirus (sh-FTO, mCherry), AKT3-expressing lentivirus (AKT3-OE, ZSGreen) or FTO knockdown lentivirus (sh-FTO + AKT3-OE) were measured via qRT‒PCR analyses. **B** The protein levels of FTO and AKT3 in KYSE150 cells infected with FTO knockdown lentivirus (sh-FTO), FTO knockdown plus AKT3-expressing lentivirus (sh-FTO + AKT3-OE) and AKT3-expressing lentivirus (AKT3-OE) were measured via western blot analyses. **C** CCK-8 assays every 24 h showed that FTO knockdown (sh-FTO) inhibited the proliferation of KYSE150 cells, while AKT3 overexpression (AKT3-OE) or FTO knockdown combined with AKT3 overexpression (sh-FTO + AKT3-OE) restored FTO function. **D** The wound healing ability of KYSE150 cells with FTO knockdown lentivirus (sh-FTO), AKT3-expressing lentivirus (AKT3-OE) or FTO knockdown plus AKT3-expressing lentivirus (sh-FTO + AKT3-OE) for 48 h was recorded (left), and the data were quantitatively analyzed (right). **E** FTO knockdown in KYSE150 cells decreased migration compared to that of the negative control (sh-NC), while AKT3 overexpression (AKT3-OE) or FTO knockdown combined with AKT3 overexpression (sh-FTO + AKT3-OE) restored FTO function. **F** FTO knockdown in KYSE150 cells decreased invasion compared to that of the negative control (sh-NC), while AKT3 overexpression (AKT3-OE) or FTO knockdown combined with AKT3 overexpression (sh-FTO + AKT3-OE) restored FTO function. **G** FTO knockdown in KYSE150 cells decreased colony formation compared to that in negative control cells (sh-NC), while AKT3 overexpression (AKT3-OE) or FTO knockdown combined with AKT3 overexpression (sh-FTO + AKT3-OE) restored FTO function

To further investigate the correlation between FTO and AKT3 in esophageal cancer tumorigenesis, we overexpressed AKT3 in KYSE150 cells and performed an additional FTO knockdown. As shown in Fig. 5A, compared to that in cells transfected with the AKT3-overexpressing vector (AKT3-OE), which increased AKT3 mRNA expression ~43.5-fold, AKT3 expression in FTO-silenced KYSE150 cells transfected with the AKT3-overexpressing vector (sh-FTO + AKT3-OE) increased ~15.3-fold, which largely abrogated the upregulation of AKT3 mRNA (Fig. 5A). These results also support the notion that FTO-regulated m^6^A demethylation promotes AKT3 mRNA stability. As a result, AKT3 protein levels were also upregulated in AKT3-OE and sh-FTO + AKT3-OE KYSE150 cells, but were slightly lower in sh-FTO + AKT3-OE cells (Fig. 5B). To test whether AKT3 could reverse the effect of FTO knockdown, we tested the proliferation ratio of KYSE150 cells using CCK-8 assays. The results showed that FTO knockdown decreased the proliferation ratio, which was restored by AKT3 overexpression in sh-FTO + AKT3-OE cells (Fig. 5C). Similarly, wound healing, migration, invasion and colony formation assays showed that the effects of FTO knockdown in KYSE150 cells could also be restored by AKT3 overexpression (Fig. 5D–G). These results also indicated that FTO-regulated m^6^A demethylation of AKT3 is associated with the tumorigenesis and metastasis of esophageal cancer cells.

### YTHDF1 maintains AKT3 mRNA stability in a m^6^A-dependent manner

**Fig. 6:**
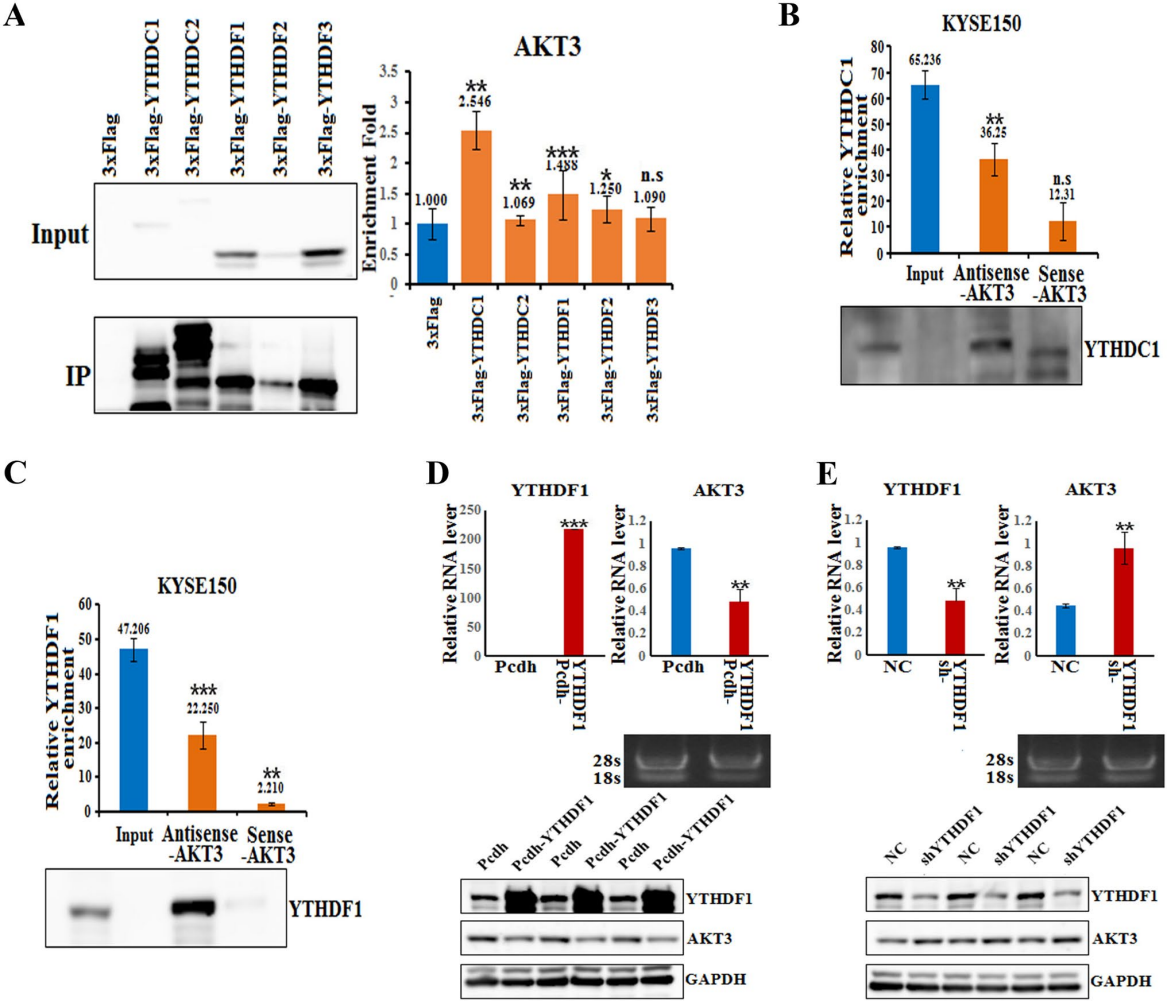
Verification of the interaction of the AKT3 gene with an m.^6^A reader. **A** RIP assays in KYSE150 cells using 3xFlag, 3xFlag-YTHDC1, 3xFlag-YTHDC2, 3xFlag-YTHDF1, 3xFlag-YTHDF2 and 3xFlag-YTHDF3 plasmids and an anti-Flag antibody. The western blots on the left show that AKT3 interacts with YTHDC1 and YTHDF1 in KYSE150 cells. The expression of AKT3 was analyzed by real-time PCR, and the results of the RIP assays are shown atthe top right. n.s., not statistically significant; *, *p* value < 0.05;**, *p* value < 0.01; ***, *p* value < 0.001. **B** Pull-down assays in KYSE150 cells were performed via transfection of biotinylated AKT3 sense probe and antisense probe (50 μl of streptavidin beads were washed once with RPD buffer, after which 10 μl of sense beads and 10 μl of antisense probe were added and incubated overnight at 4 ℃). Then, the cells were collected for the biotin-based pull-down assay. YTHDC1 expression levels were analyzed by real-time PCR analysis and western blotting. n.s., not statistically significant; **, *p* value < 0.01. **C** Pull-down assays in KYSE150 cells were performed via transfection of biotinylated AKT3 sense probe and antisense probe (50 μl of streptavidin beads were washed once with RPD buffer, after which 10 μl of sense beads and 10 μl of antisense probe were added and incubated overnight at 4 ℃). Then, the cells were collected for the biotin-based pull-down assay. YTHDF1 expression levels were analyzed by real-time PCR and western blotting. **, *p* value < 0.01; ***, *p* value < 0.001. **D** Real-time PCR analysis at the top shows the expression of YTHDF1 and AKT3 in the YTHDF1-overexpressing cells (YTHDF1-OE), and agarose electrophoresis of the PCR products is also shown. Western blot analysis at the bottom shows the expression of YTHDF1 and AKT3 in cells overexpressing YTHDF1 (YTHDF1-OE). **E** Real-time PCR analysis at the top shows the expression of YTHDF1 and AKT3 in cells with YTHDF1 knockdown (sh-YTHDF1), and agarose electrophoresis of the PCR products is also shown. Western blot analysis at the bottom shows the expression of YTHDF1 and AKT3 after YTHDF1 knockdown (sh-YTHDF1)

Previous studies have identified two major families of m^6^A “readers” that might play a specific role in controlling the fate of methylated mRNAs, the YTH family and the IGF2BP family. To identify the specific m^6^A readers of AKT3 and determine the m^6^A-dependent mechanism of AKT3 regulation, we performed FLAG RNA pull-down assays in KYSE150 cells to screen for AKT3-related m^6^A readers. Notably, YTHDC1 and YTHDF1, but not other members of the YTH family, specifically bind to the full-length AKT3 transcripts in KYSE150 cells (Fig. 6A). Biotin-based pull-down assays also confirmed the direct interactions of AKT3 mRNA with both YTHDC1 and YTHDF1, indicating a potential positive regulatory mechanism (Fig. 6B, C).

To further test the role of YTHDF1 in regulating AKT3 stability, we inhibited or increased the expression of YTHDF1 in KYSE150 cells. As a result, AKT3 mRNA and protein expression were decreased upon the overexpression of YTHDF1 in KYSE150 cells. Moreover, AKT3 mRNA and protein expression were upregulated after siRNA-mediated inhibition of YTHDF1 in KYSE150 cells (Fig. 6D, E). Taken together, our results suggested that the methylated AKT3 transcripts might be directly recognized by YTHDF1, which maintains the stability of the AKT3 transcripts.

### FTO and AKT3 promote esophageal cancer progression in vivo

**Fig. 7:**
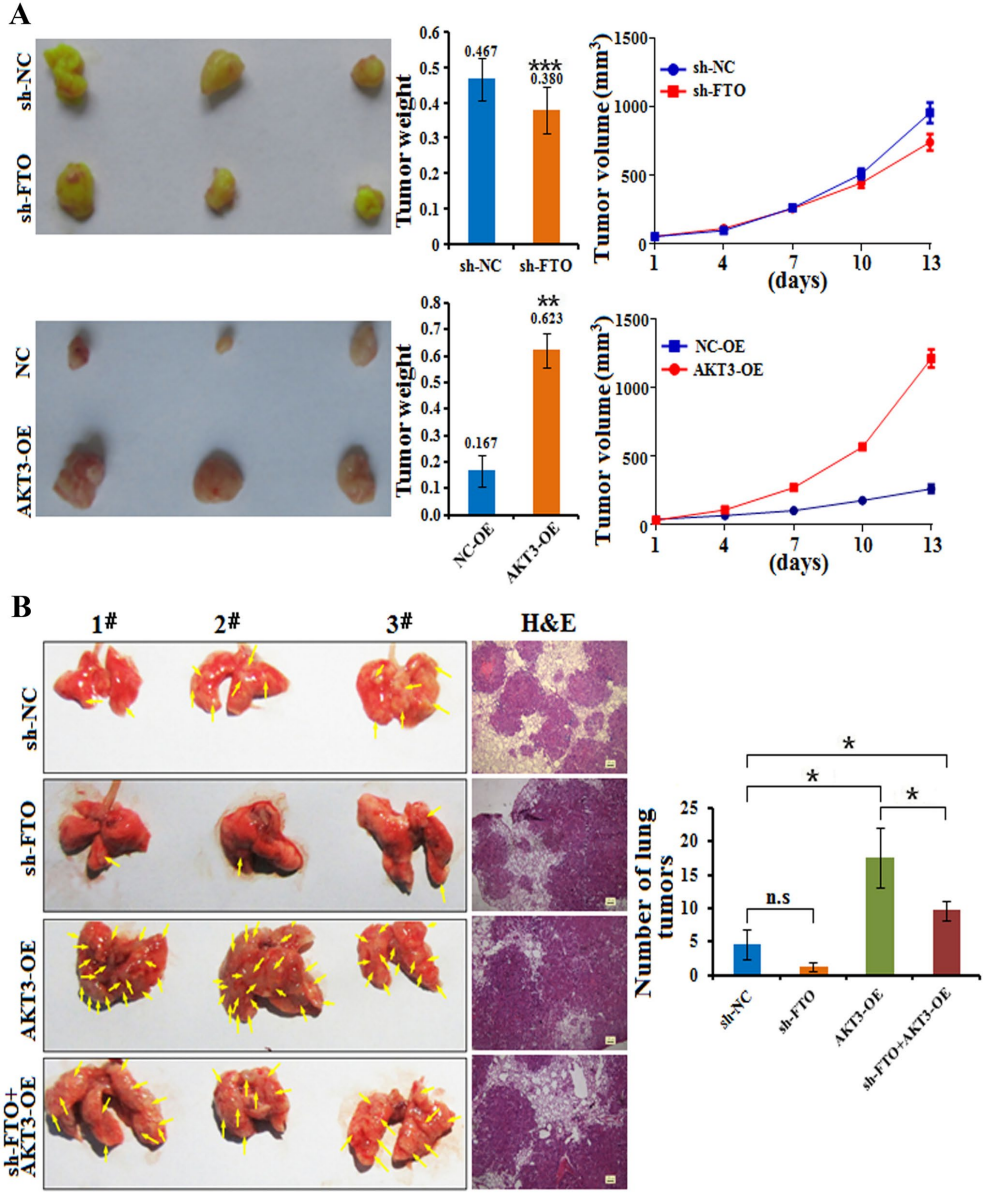
FTO and AKT3 promote esophageal cancer invasion in vivo. **A** Cells with stable FTO knockdown or stable FTO expression were subcutaneously injected at two time points on the back of each nude mouse. Representative images of the tumors on the 13th day of tumor formation, tumor weight and tumor growth curve of xenografts generated by FTO knockdown (sh-FTO) and AKT3-expressing lentivirus (AKT3-OE) versus the negative controls sh-NC and NC-OE, respectively (*n* = 3 for each group, **p < 0.01; ***p < 0.001 by Student’s *t* test). **B** For the in vivo lung metastasis model, cells with stable FTO knockdown or stable FTO expression were subcutaneously injected into nude mice via the tail vein. WT (wild type), sh-FTO, AKT3-OE and sh-FTO + AKT3-OE KYSE150 cells were injected (1 × 10.^6^ per mouse, *n* = 3 for each group). Six weeks later, the mice were killed, and metastatic lung tumors were analyzed. Representative images of metastatic lung tumors and H&E staining results are shown (left), and the number of lung tumors was quantitatively analyzed (right)

To test the potential role of FTO and AKT3 in esophageal cancer biogenesis in vivo, we injected sh-FTO- or AKT3-overexpressing KYSE150 cells subcutaneously into nude mice. Then, the mice were killed when the tumor volume was approximately 1000 mm^3^ in each group. Compared to those in the control groups, the weights of the sh-FTO-transfected KYSE150 cells were significantly lower, whereas the weights of the tumors were greater in the AKT3-OE KYSE150 cells (Fig. 7A). To further determine the impacts of m^6^A methylation on in vivo metastasis, sh-FTO, AKT3-OE or sh-FTO & AKT3-OE KYSE150 cells were injected into nude mice via the tail vein to analyze lung colonization. As shown in Fig. 7B, the number of lung tumors derived from FTO knockdown KYSE150 cells did not significantly change; however, AKT3-OE or sh-FTO & AKT3-OE significantly promoted the number of lung tumors compared with that in control cells, suggesting that AKT3 overexpression promoted tumor metastasis in vivo. These results suggested that FTO and AKT3 are involved in esophageal cancer progression in vivo.

## Discussion

Increasing evidence indicates that m^6^A modifications in mRNAs are involved in numerous biological functions and in the progression of cancer (Lin et al. 2019; Cui et al. 2017). In this study, we demonstrated that m^6^A modifications in mRNAs can regulate the progression of esophageal cancer. Currently, only two proteins have been found to have demethylase activity via m^6^A modification. On the basis of our previous finding that ALKBH5 expression is positively correlated with the prognosis of esophageal cancer patients, while for the other m^6^A demethylase protein FTO, we found that it can promote the proliferation and invasion of esophageal cancer cells but is not related to the prognosis of esophageal cancer patients (Kong et al. 2023). Based on these results, we investigated the correlation between the combined effect of FTO and multiple m^6^A regulatory proteins and the prognosis of esophageal cancer patients and found that an increase in the FTO/METTL14 ratio can lead to poor prognosis. It was speculated that FTO and METTL14 have antagonistic effects. Changes in FTO and METTL14 levels largely affect the in vitro proliferation, migration, and invasion of cancer cells. Further investigations identified AKT3 asa target of FTO. In particular, AKT3 is a phosphatidylinositol-3-kinase, and the protein kinase B family is a key element of the PI3K/AKT signaling pathway. The AKT pathway was found to regulate many hallmarks of cancer and the metastatic cascade in breast cancer (Altomare and Testa 2005; Castaneda et al. 2010; Nicholson and Anderson 2002). In addition, much effort has been made to develop targeted therapies targeting AKT signaling in breast cancer (Barnett et al. 2005; Hernandez-Aya and Gonzalez-Angulo 2011; Araki and Miyoshi 2018). Thus, the PI3K/AKT pathway is a promising target for cancer therapy owing to the high frequency of dysregulation of this pathway in human breast cancer (Lopez-Knowles et al. 2010). Here, we showed that AKT3 is involved in the progression of esophageal cancer, which provides the basis for further targeting the AKT3 pathway for clinical treatment of esophageal cancer.

However, the role of mRNA modification in controlling cancer progression has not been fully elucidated. As the first characterized m^6^A demethylase, FTO has been reported to regulate the tumorigenesis of different types of cancers. FTO was found to enhance leukemic oncogene-mediated cell transformation and leukemogenesis by reducing the m^6^A levels of its targets (Li et al. 2017b). In addition, pharmaceutical inhibition of FTO by a chemical inhibitor inhibits tumor progression and significantly prolongs the life of glioblastoma stem cell-transplanted mice (Cui et al. 2017). On the other hand, METTL14, which is the methyltransferase of m^6^A mRNA, has several functions in cancer cells, such as regulating leukemogenesis and proliferation of hematopoietic stem/progenitor cells (HSPCs) (Weng et al. 2018). Targeting METTL14, especially in combination with differentiation inducers, may be an effective new therapeutic strategy for the treatment of AML. In addition, METTL14 and METTL3 form a stable heterodimeric core complex that plays a role in cell m^6^A deposition and can inhibit metastatic potential by regulating primary microRNA126 treatment in a m^6^A-dependent manner (Ma et al. 2017). In this study, we elucidated the link between the regulation of AKT3 mRNA m^6^A methylation by FTO and METTL14. We found that downregulation of FTO or overexpression of METTL14 had similar effects on multiple aspects of esophageal cancer progression, including migration, invasion, proliferation, and tumorigenesis, which also suggested that FTO function could be restored by METTL14 in esophageal cancer. Our results describe the roles of m^6^Aand FTO in cancer progression and provide a basis for the development of therapeutic strategies against esophageal cancer metastasis. Notably, the METTL14/FTO/AKT3 signaling network might have other normal functions in addition to influencing tumorigenesis in esophageal cancer. However, additional investigations are needed to clarify the detailed regulatory mechanism of these players in cellular functions.

The m^6^A modification modulates all stages of the life cycle, such as RNA processing, nuclear export, and translation (Zhao et al. 2017b, 2018). For example, m^6^A modification can promote the alkenylation of RNA through the first characterized m^6^A “reader” protein YTHDF2, thereby triggering mRNA degradation (Wang et al. 2014). Here, we showed that the stability of AKT3 mRNA transcripts is enhanced by the reader protein YTHDF1 (Shi et al. 2019). Previous studies have shown that YTHDF1 is linked to the progression of various cancers, including non-small cell lung cancer (Bai et al. 2019), colorectal carcinoma (Bai et al. 2019), and ovarian cancer (Liu et al. 2020b). In our study, we found that the knockdown of YTHDF1 decreased AKT3 at both the mRNA and protein levels, whereas YTHDF1 overexpression significantly enhanced AKT3 at the mRNA and protein levels. These data support that AKT3 is the direct target of YTHDF1 in esophageal cancer. The results indicated that YTHDF1 might regulate the transcription and translation of AKT3. However, the detailed mechanism by which YTHDF1 regulates AKT3 expression needs further investigation.

## Conclusions

We provide a large amount of in vitro and in vivo evidence that m^6^A modification can regulate the progression of esophageal cancer by promoting the growth, survival and invasion of cancer cells. We found that the m^6^A methyltransferase METTL14 is also involved in FTO-regulated m^6^A modification and acts in concert with FTO to regulate AKT3 methylation in the tumorigenesis and metastasis of esophageal cancer. Importantly, we revealed that FTO operates through a regulatory network of m^6^A modifications that involves METTL14, YTHDF1 and AKT3 signaling, providing the first insight into the mechanism of FTO-mediated esophageal cancer progression.

## Supplementary Information

Below is the link to the electronic supplementary material.

Figure S1 FTO expression in normal esophageal tissue and esophageal cancer patients. A and B. Prognostic signatures based on ALKBH5 and FTO in predicting OS in patients. The figure contains three parts: [1] survival differences estimated by Kaplan‒Meier survival curve; [2] number of patients in different groups; and [3] number censored at different times. C. Real-time PCR analysis and western blotting analysis of FTO expression in five esophageal cancer cell lines and one normal esophageal cell line. D. FTO was upregulated in esophageal cancer tissues compared with normal tissues (GEPIA data, red box for tumor tissue, n = 182; gray box for normal tissue, n = 286). E. Western blotting analysis of FTO expression in three paired esophageal cancer primary tumor samples. F. Representative image of immunohistochemical staining for FTO in 400x-thick magnified esophageal squamous cell carcinoma (ESCC) tissues and paired normal tissues from human samples (above).Immunohistochemical expression of FTO in ESCC tumor tissue and paired paracancerous tissue (PT) samples was quantitatively analyzed using IMAGE-PRO PLUS 6.0 software (below). Scale bar=50 μm, n=28. (TIF 16700 KB)

Figure. S2 A. The protein levels of E-cadherin and FTO in KYSE150 cells with FTO knockdown versus the negative control (sh-NC) measured by western blot analyses. B. The protein levels of Vimentin and AKT3 in KYSE150 cells with AKT3 overexpression versus the negative control (NC-OE) measured by western blot analyses. (TIF 7984 KB)

Supplementary file3 (XLSX 12 KB)

Supplementary file4 (XLSX 13 KB)

Supplementary file5 (DOCX 738 KB)

## Data Availability

The data presented in the study are deposited in the SRAdatabase (accession number PRJNA889200).

## Abbreviations

m^6^A: *N*^6^-Methyladenosine
FTO: Fat mass- and obesity-associated protein
AKT3: AKT serine/threonine kinase 3
METTL14: Methyltransferase-like 14
WTAP: Wilms tumor 1-associated protein
ALKBH5: Alkylation repair homolog protein 5
YTHDF1: YTH *N*^6^-methyladenosine RNA-binding protein 1
YTHDF2: YTH *N*^6^-methyladenosine RNA-binding protein 2
IGF2BP1: Insulin-like growth factor 2 mRNA-binding protein 1
IGF2BP2: Insulin-like growth factor 2 mRNA-binding protein 2
E-cad: E-cadherin
VIM: Vimentin
MMP2: Matrix metallopeptidase 2
ActD: Actinomycetes D
MeRIP-seq: Methylated RNA immunoprecipitation
RNA-seq: Transcriptomic RNA sequencing
IHC: Immunohistochemical
RIP: RNA immunoprecipitation
RT–qPCR: Quantitative real-time PCR
SDS–PAGE: Sodium dodecyl sulfate–polyacrylamide gel electrophoresis
3′UTR: Three prime untranslated region
CDS: Coding sequence
WT: Wild type
PVDF: Polyvinylidene fluoride
TCGA: The Cancer Genome Atlas
USTC: University of Science and Technology of China
