# Supplementary figures and images for: The antagonistic effect of FTO on METTL14 promotes AKT3 m6A demethylation and the progression of esophageal cancer
Source: J Cancer Res Clin Oncol. 2024 Mar 15;150(3):131. doi: 10.1007/s00432-024-05660-2 (PMC10943165; doi:10.1007/s00432-024-05660-2)

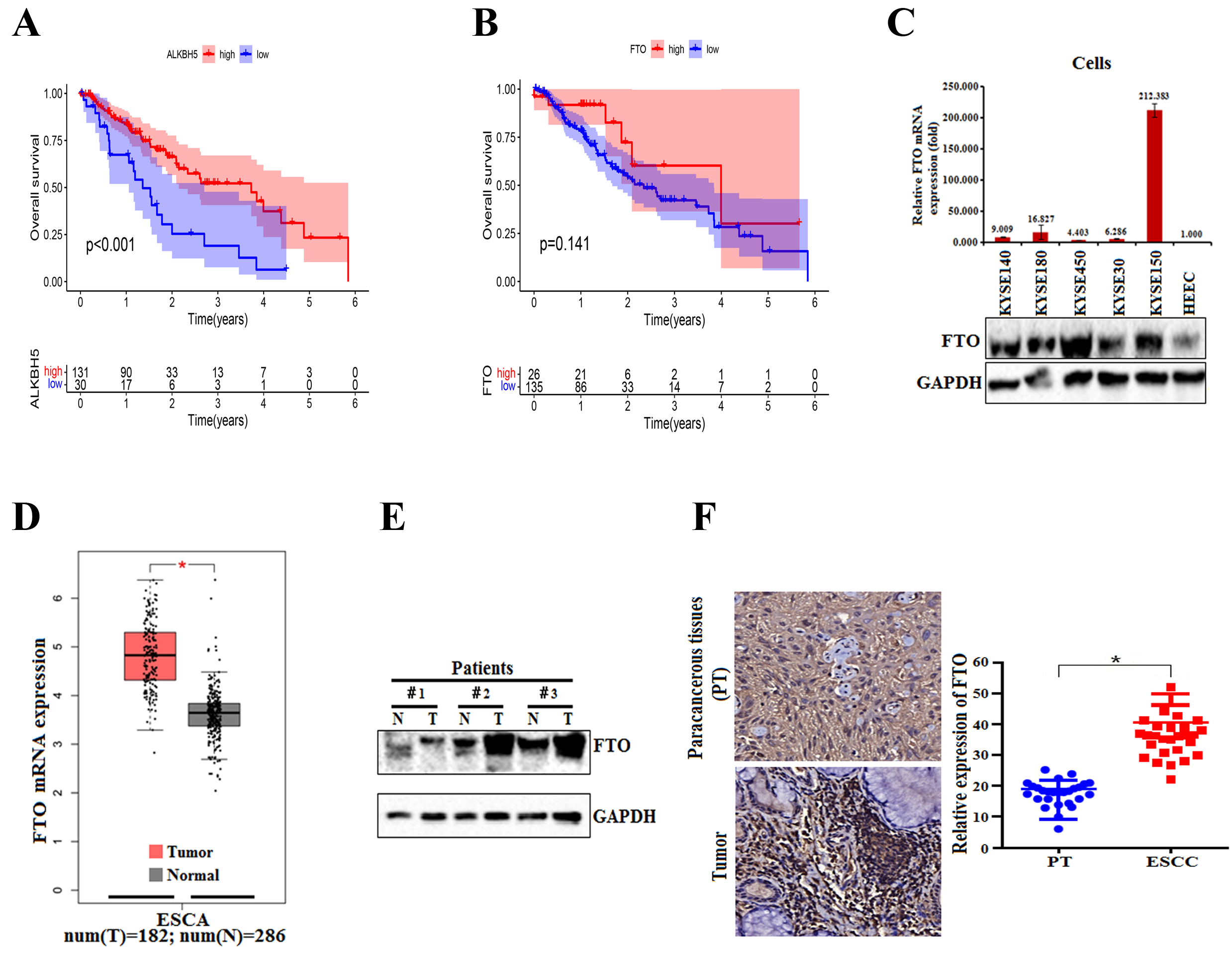

Supplement: Supplementary file 1 — Figure S1 FTO expression in normal esophageal tissue and esophageal cancer patients. A and B. Prognostic signatures based on ALKBH5 and FTO in predicting OS in patients. The figure contains three parts: [1] survival differences estimated by Kaplan‒Meier survival curve; [2] number of patients in different groups; and [3] number censored at different times. C. Real-time PCR analysis and western blotting analysis of FTO expression in five esophageal cancer cell lines and one normal esophageal cell line. D. FTO was upregulated in esophageal cancer tissues compared with normal tissues (GEPIA data, red box for tumor tissue, n = 182; gray box for normal tissue, n = 286). E. Western blotting analysis of FTO expression in three paired esophageal cancer primary tumor samples. F. Representative image of immunohistochemical staining for FTO in 400x-thick magnified esophageal squamous cell carcinoma (ESCC) tissues and paired normal tissues from human samples (above).Immunohistochemical expression of FTO in ESCC tumor tissue and paired paracancerous tissue (PT) samples was quantitatively analyzed using IMAGE-PRO PLUS 6.0 software (below). Scale bar=50 μm, n=28. (TIF 16700 KB) [file 432_2024_5660_MOESM1_ESM.tif]

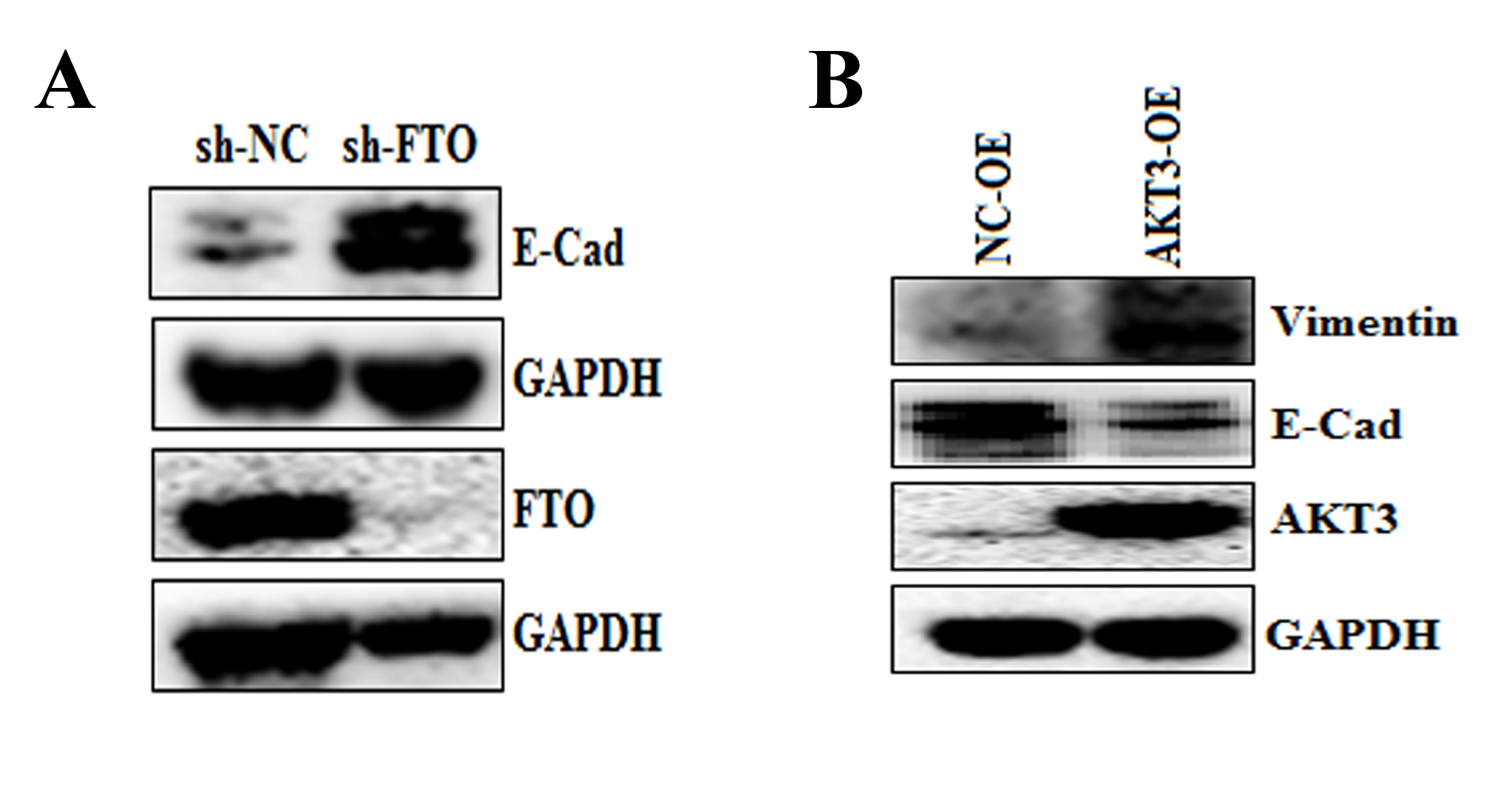

Supplement: Supplementary file 2 — Figure. S2 A. The protein levels of E-cadherin and FTO in KYSE150 cells with FTO knockdown versus the negative control (sh-NC) measured by western blot analyses. B. The protein levels of Vimentin and AKT3 in KYSE150 cells with AKT3 overexpression versus the negative control (NC-OE) measured by western blot analyses. (TIF 7984 KB) [file 432_2024_5660_MOESM2_ESM.tif]
