# Supplementary material for: The antagonistic effect of FTO on METTL14 promotes AKT3 m6A demethylation and the progression of esophageal cancer
Source: J Cancer Res Clin Oncol. 2024 Mar 15;150(3):131. doi: 10.1007/s00432-024-05660-2 (PMC10943165; doi:10.1007/s00432-024-05660-2)
Supplement: Supplementary file 5 — Supplementary file5 (DOCX 738 KB) [file 432_2024_5660_MOESM5_ESM.docx]

# Supplementary Figures:


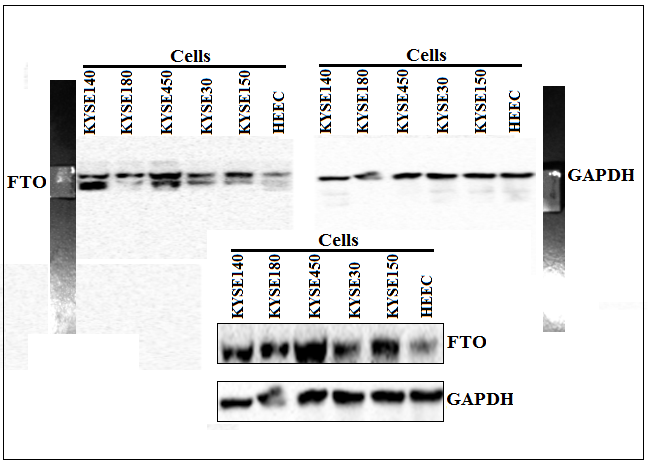


# Fig S1. The full-length gels of the Figure1D, western analyses used in the revised manuscript.


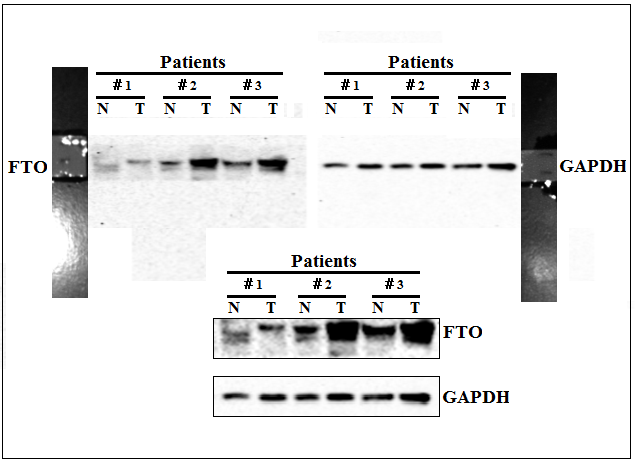


# Fig S2. The full-length gels of the Figure1E, western analyses used in the revised manuscript.


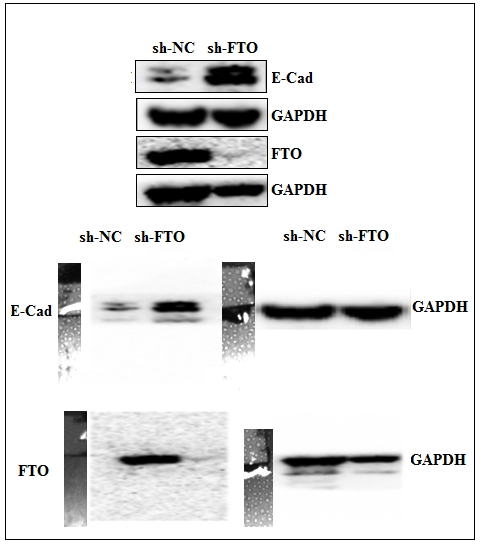


# Fig S3. The full-length gels of the Figure2D, western analyses used in the revised manuscript.


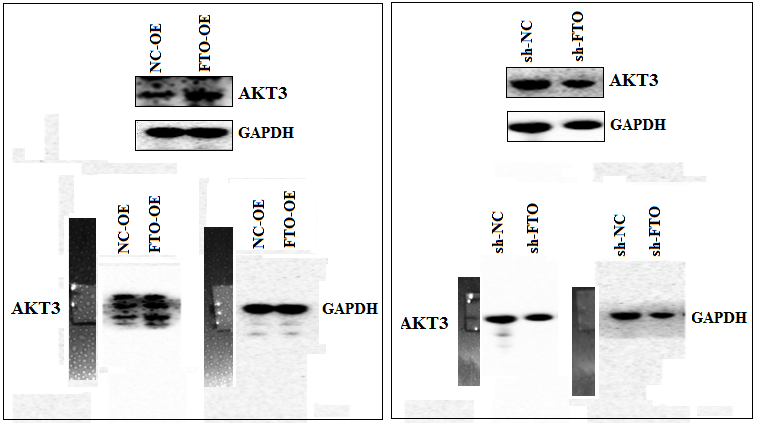


# Fig S4. The full-length gels of the Figure5A and 5B, western analyses used in the revised manuscript.


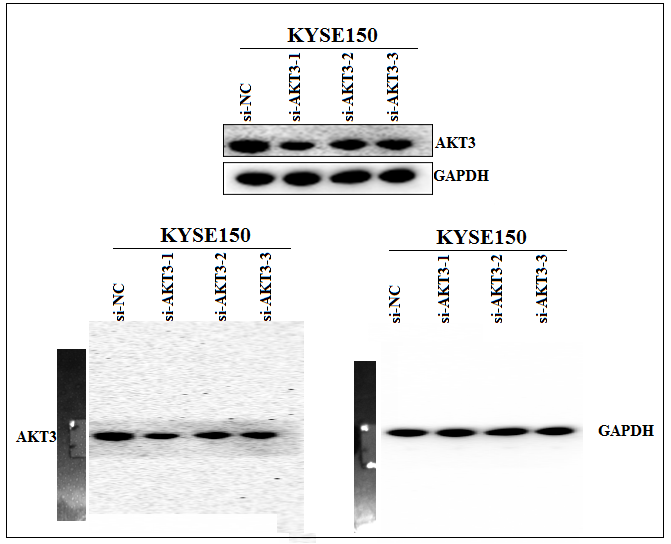


# Fig S5. The full-length gels of the Figure5D, western analyses used in the revised manuscript.


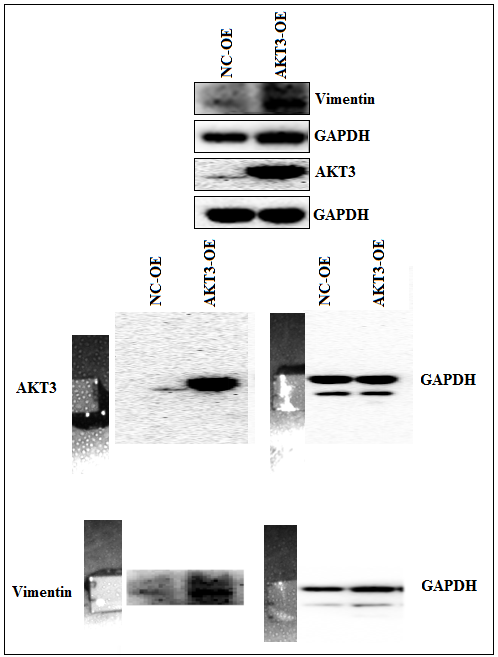


# Fig S6. The full-length gels of the Figure5E, western analyses used in the revised manuscript.


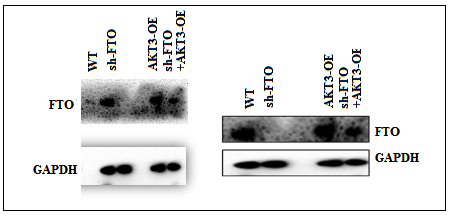


# Fig S6. The full-length gels of the Figure6B, western analyses used in the revised manuscript.


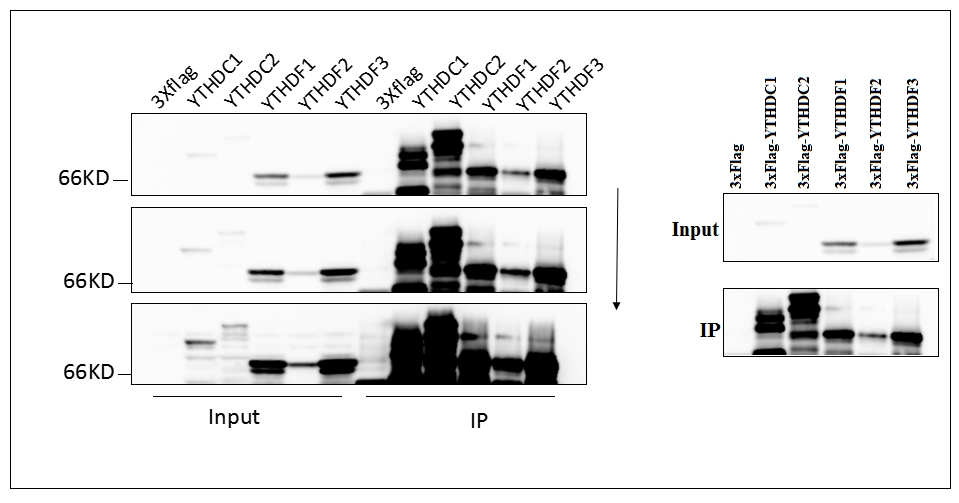


# Fig S7. The full-length gels of the Figure7A, western analyses used in the revised manuscript.


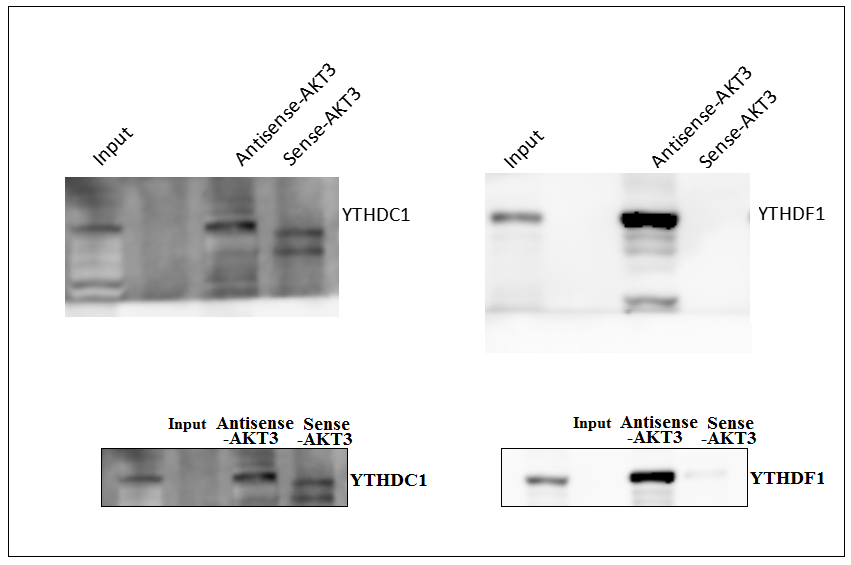


# Fig S8. The full-length gels of the Figure7B, western analyses used in the revised manuscript.


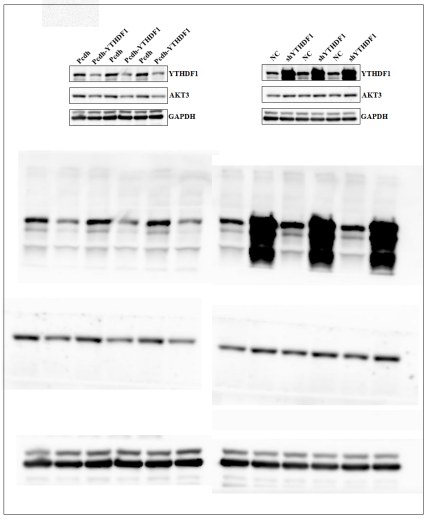


# Fig S9. The full-length gels of the Figure7F and 7G, western analyses used in the revised manuscript.
